# Proteases, Mucus, and Mucosal Immunity in Chronic Lung Disease

**DOI:** 10.3390/ijms22095018

**Published:** 2021-05-09

**Authors:** Michael C. McKelvey, Ryan Brown, Sinéad Ryan, Marcus A. Mall, Sinéad Weldon, Clifford C. Taggart

**Affiliations:** 1 Airway Innate Immunity Research (AiiR) Group, Wellcome-Wolfson Institute for Experimental Medicine, Queen’s University Belfast, Belfast BT9 7BL, UK; m.mckelvey@qub.ac.uk (M.C.M.); rr.brown@qub.ac.uk (R.B.); sryan18@qub.ac.uk (S.R.); s.weldon@qub.ac.uk (S.W.); 2 Department of Pediatric Respiratory Medicine, Immunology and Critical Care Medicine, Charité—Universitätsmedizin Berlin, 13353 Berlin, Germany; marcus.mall@charite.de; 3 Berlin Institute of Health (BIH), 10178 Berlin, Germany; 4 German Center for Lung Research (DZL), 35392 Gießen, Germany

**Keywords:** chronic lung disease, proteases, antiproteases, mucus, muco-obstructive lung disease, mucociliary clearance, mucosal immunity, inflammation

## Abstract

Dysregulated protease activity has long been implicated in the pathogenesis of chronic lung diseases and especially in conditions that display mucus obstruction, such as chronic obstructive pulmonary disease, cystic fibrosis, and non-cystic fibrosis bronchiectasis. However, our appreciation of the roles of proteases in various aspects of such diseases continues to grow. Patients with muco-obstructive lung disease experience progressive spirals of inflammation, mucostasis, airway infection and lung function decline. Some therapies exist for the treatment of these symptoms, but they are unable to halt disease progression and patients may benefit from novel adjunct therapies. In this review, we highlight how proteases act as multifunctional enzymes that are vital for normal airway homeostasis but, when their activity becomes immoderate, also directly contribute to airway dysfunction, and impair the processes that could resolve disease. We focus on how proteases regulate the state of mucus at the airway surface, impair mucociliary clearance and ultimately, promote mucostasis. We discuss how, in parallel, proteases are able to promote an inflammatory environment in the airways by mediating proinflammatory signalling, compromising host defence mechanisms and perpetuating their own proteolytic activity causing structural lung damage. Finally, we discuss some possible reasons for the clinical inefficacy of protease inhibitors to date and propose that, especially in a combination therapy approach, proteases represent attractive therapeutic targets for muco-obstructive lung diseases.

## 1. Introduction

Proteases are enzymes that catalyse the hydrolysis of peptide bonds within proteins, facilitating their cleavage; this hydrolysis can either activate, inactivate, or modulate the activity of the target protein. The identities of the amino acid residues that form the catalytic site have been used to group human proteases into serine, cysteine, matrix metallo-, aspartyl, and threonine protease classes. Within the lung, serine, cysteine and metalloproteases have received the most attention to date [1,2]. In healthy cells and tissues, both intracellular and extracellular protease activity is well managed by regulation at the transcriptional and translational levels, as well as by inhibitory pro-domains, modulatory factors (such as pH), and antiproteases at the protein level. However, higher-than-normal protease levels and excessive protease activity are recognised as hallmarks in chronic lung diseases (CLDs) and we continue to gain a greater appreciation of how the protease burden contributes to pathology [3,4,5]. This review will focus on the contributions of proteases at the airway mucosal surface, including how they influence important aspects of airway function including mucus characteristics, mucociliary clearance (MCC) and immune cell recruitment and function.

Lung health is a product of many environmental and host factors, including exposure to toxins, particulates or pathogens, the mounting of appropriate immune responses to such stimuli, efficient ventilation mechanics and effective gas exchange. The mucosal surfaces of the airways are important interfaces for environmental and host factors, and alterations at this interface are a common feature in patients with CLD. The mucosal surface of the airway is composed of epithelial cells, many of which are ciliated, and is coated with a thin apical layer of mucus, resident and recruited immune cells, and the inhaled contents of the airway lumen. In many CLDs, the most obvious clinical symptoms are related to airway mucus, its excessive production, and an inability to clear it. MCC is a vital feature of the innate immune system in the airways [6,7]. A number of processes are essential to maintain effective MCC including regulation of ion channel activity, ciliary beat frequency (CBF), mucin expression and secretion and mucus viscosity [8]. Mucus is a hydrogel composed of water, salts, large mucin polymers, non-mucin proteins, lipids, and cellular debris [9,10]. Under normal conditions, water makes up 97–98% of mucus, producing a loose and mobile gel that ably protects the airway surface from inhaled pathogens and toxins, which are removed from the airways by ciliary beat and cough. However, in many CLDs, and especially the so-called ‘muco-obstructive’ lung diseases (chronic obstructive pulmonary disease (COPD), cystic fibrosis (CF), primary ciliary dyskinesia (PCD) and non-CF bronchiectasis), mucus composition is radically altered, producing a hyper-concentrated mucus layer [10,11,12,13]. The osmotic pressure of this hyper-concentrated mucus layer can exceed that of the subjacent periciliary layer, causing compression and flattening of the cilia, resulting in impaired ciliary beating and reduced mucus clearance. This leads to mucostasis and the build-up of mucus plaques and plugs in the airway lumen, producing muco-obstructive lung disease. The inciting causes of these original changes in the airways, mucus composition and MCC, vary between the different muco-obstructive lung diseases (environmental factors, recurrent infection, genetic mutations to ion channels etc.), but they share pathological mechanisms, many of which are mediated or modulated by proteases.

## 2. Proteases and Mucus

### 2.1. Proteases and Ion Transport

Ion channel activity is critical to maintain the airway surface liquid (ASL) at an appropriate height for effective MCC [14]. This is primarily achieved through the regulation of Cl^−^ secretion and Na^+^ absorption via the chloride channel, cystic fibrosis transmembrane conductance regulator (CFTR) and the sodium channel, epithelial sodium channel (ENaC), respectively [15]. Defects in airway ion transport result in the development of muco-obstructive lung diseases, most notably with the loss of CFTR function in CF [16,17]. The role of proteases in regulating airway ion transport has largely focused on the activation of ENaC. ENaC undergoes maturation in the Golgi through the removal of an inhibitory peptide in its α-subunit by furin-type convertases [18]. These processed channels are classed as having intermediate open probability. However, release of a second inhibitory peptide from the γ-subunit at the plasma membrane can result in ENaC channels with a high open probability. This secondary cleavage is under the regulation of extracellular proteases. A number of proteases have been shown to cleave γ-ENaC, increasing the open probability of ENaC; these include serine proteases such as channel activating protease (CAP)-1, neutrophil elastase (NE), trypsin, chymotrypsin, prostasin and transmembrane protease serine 4 (TMPRSS4), as well as the cysteine proteases cathepsin B (CTSB) and cathepsin S (CTSS) [19,20,21,22,23,24]. Indeed, inhibition of trypsin-like serine proteases using the synthetic inhibitor ONO-3403 resulted in marked improvements in pulmonary dysfunction and emphysema in a murine model of CLD, indicating the importance of this ENaC-regulatory process [25]. Bacterial proteases including alkaline protease released from *Pseudomonas aeruginosa* also cleave and activate ENaC [26]. This activation of ENaC by both human and bacterial proteases is highly relevant in CLD, particularly where bacterial colonisation is prevalent. Increased ENaC activity is associated with severity in COPD and was shown to cause muco-obstructive lung disease in mice [27,28,29]. Conversely, decreased ENaC activity in patients with pseudohypoaldosteronism improved mucus clearance rates [30].

Protease-dependent regulation of CFTR has also been observed. Unlike ENaC, CFTR is not activated by proteolytic cleavage at the plasma membrane. However, the level of CFTR present at the cell surface is under the regulation of the cysteine protease calpain, which cleaves mature CFTR at the plasma membrane, allowing it to be internalised in vesicles for degradation [31]. Increased calpain activity is observed in CF, resulting in instability and reduced cell surface retention of CFTR that reaches the plasma membrane [32,33,34]. NE released from activated neutrophils, which are abundant in the chronically inflamed lung, also induces proteolysis and internalisation of CFTR on airway epithelial cells via the induction of this calpain-dependent degradation pathway [35]. Protease-dependent CFTR dysfunction may be important in chronic lung conditions beyond CF [36]. Indeed, CFTR function is associated with severity of emphysema in COPD [37]. There is also increasing evidence that loss of CFTR function resulting from exposure to cigarette smoke may promote smoking-associated lung disease [38,39]. These regulatory mechanisms are highly valuable in allowing dynamic changes in salt and water reabsorption and secretion in response to changing environments. However, in muco-obstructive lung disease, with a loss in protease/antiprotease balance, increased protease activity could lead to excessive Na^+^ absorption and/or loss of Cl^−^ transport, with associated dehydration of the airways. Acidification of the ASL as a result of CFTR dysfunction may also play a part, stimulating the activity of cysteine cathepsins and further upregulating ENaC activity [40]. These studies highlight important roles for proteases in maintaining airway ion balance. Additionally, they suggest that targeting proteases may aid in regulating and maintaining effective ion transport and ASL height in muco-obstructive lung disease. The majority of research into protease regulation of airway ion channel activity has used cell culture models or *Xenopus laevis* oocytes. As such, there is currently little evidence for the direct therapeutic benefit of using protease inhibitors to alter ion channel activity in muco-obstructive lung disease, and this should be an area for future study. 

### 2.2. Proteases and Ciliary Function

Cilia lining the epithelium of the airways play an important role in driving MCC; beating in a synchronised fashion, they facilitate removal of pathogens and debris trapped in the mucus layer. In the large airways, ciliated cells typically make up ~80% of the epithelium [41]. In muco-obstructive lung disease, ciliary beating is hindered by airway dehydration and increased mucus viscosity. Furthermore, as a result of goblet cell hyperplasia, the percentage of ciliated cells in the airway epithelium can drop as low as 20% [42]. The importance of proper ciliary function is evident in PCD, where abnormal ciliary beating leads to mucus plugging and chronic infection [43]. Protease activity contributes both directly and indirectly to the maintenance of ciliary stability and function. Optimal CBF is required for MCC and is regulated by a number of factors including cyclic adenosine monophosphate (cAMP)-dependent phosphorylation, intracellular Ca^2+^ levels and pH [44,45,46]. Ciliary beating is powered by molecular motors known as dyneins, which induce a series of contractions along the nine doublet microtubules making up the extracellular cilia axoneme and in doing so, produce the ciliary beat [47]. As such, dynein is an essential component of motile cilia. Cleavage of dynein by the serine proteases trypsin and subtilisin results in a loss of ciliary motility [48]. In addition to these human proteases, bacterial proteases are also capable of disrupting airway cilia by the same mechanism [48]. NE has also been shown to reduce CBF in vitro in human nasal bronchial epithelial cells. However, this effect was only observed in cells that were treated with high concentrations of NE [49]. Reductions in CBF in this case were likely a result of damage to the ciliated cells rather than a mechanistic alteration to ciliary beat, as histological examination revealed epithelial disruption while the number and ultrastructure of the cilia appeared normal [49]. This is still a significant finding because along with goblet cell metaplasia, this protease-dependent cell damage may contribute to the significant reduction in ciliated cells in the diseased airways. In contrast, while direct protease activity may lead to ciliated cell disruption, activation of protease-activated receptor (PAR)-2 by proteases secreted from airway neutrophils increases ciliary beating by 30–50% through the induction of Ca^2+^ signalling [50]. This could represent a clearance mechanism initiated during inflammation to clear inflammatory stimuli from the airways. 

In addition to altering cilia motility, proteases can also affect cilia stability. Increased intracellular calpain activity is associated with diminished formation of cilia. Cilia require anchoring to the cell cytoskeleton by a basal body from which the axoneme is assembled. Calpain targets proteins in the basal body, resulting in a loss of anchoring and failed cilia formation [51]. The specific substrate(s) of calpain in the basal body structure have not been fully elucidated, though ezrin, a protein involved in plasma membrane/actin cytoskeleton interactions, is localised to the basal body and, as a known substrate of calpain, is a likely candidate [52]. These data present a varying effect of proteases on ciliary function. Increased protease activity in the chronically inflamed lung leads to reduced ciliary stability and motility and disruption of ciliated epithelial cells. Conversely, PAR-2 signalling may increase CBF in those ciliated cells that remain intact.

### 2.3. Proteases and Mucus Properties

Sitting atop the periciliary layer is a layer of mucus that traps debris and pathogens as it gradually moves from the distal to proximal airways along the mucociliary escalator. This mucus layer consists primarily of water, but also contains large polymeric mucin glycoproteins that determine the viscoelastic properties of the mucus layer [9]. These mucins are separated into secreted and tethered mucins depending on their properties. In the airways, the predominant secreted gel-forming mucins are mucin (MUC)-5AC and MUC5B [53]. Maintaining a mucus layer with the right properties is important for effective MCC and alterations in the composition of this mucus layer are associated with the development of chronic airway diseases [54,55,56]. Regulation is achieved through the maintenance of a number of factors including mucin expression and secretion, and mucus viscosity, which is largely determined by mucus hydration and crosslinking of mucins [10,57]. 

#### 2.3.1. Mucin Expression

The role of proteases in the regulation of mucin gene expression has been examined in several studies, largely focusing on the regulation of *MUC5AC* expression, with little assessment of the regulation of *MUC5B*. This is likely a result of the current dogma that MUC5AC upregulation is the driving force behind mucus phenotypes in CLDs, while MUC5B is required for maintaining normal MCC [58]. The serine protease NE induces *MUC5AC* messenger ribonucleic acid (mRNA) and protein in airway epithelial cells (AECs) through increased mRNA stability or via a retinoic acid receptor-dependent mechanism [59,60]. Furthermore, induction of oxidative stress by NE has been shown to increase *MUC5AC* expression [61,62]. Changes in *MUC5AC* expression were not observed upon exposure of AECs to cysteine or metalloproteases in this study, suggesting these mechanisms may be specific to serine proteases [60]. However, in a separate study, a disintegrin and metalloprotease 17 (ADAM-17) and matrix metalloprotease 9 (MMP-9) induced *MUC5AC* expression through the activation of epidermal growth factor receptor (EGFR) [63]. Another serine protease, human airway trypsin-like protease (HAT) indirectly induced mucin gene expression in AECs through a similar mechanism [64]. Treatment of AECs with HAT induced expression and secretion of the EGFR ligand amphiregulin, leading to EGFR pathway activation and increased *MUC5AC* expression [64]. Interestingly, protease-mediated changed in CFTR and ENaC activity may also impact mucin production. For example, changes in these ion channels have been shown to lower intracellular Zn^2+^ concentrations by inducing alternative splicing of the zinc importer, ZIP2, which in turn drives MUC5AC hypersecretion [65].

In addition to human proteases, fungal proteases also regulate mucin expression. Notably, proteases released by *Aspergillus fumigatus*, a fungus that is highly prevalent in the early CF lung, induce *MUC5AC* expression [66,67]. A more recent study identified a Ras/Raf1/extracellular signal-regulated kinase (ERK) signalling pathway through which mucin expression was induced by fungal proteases [68]. Upregulation of MUC5AC by NE and other proteases in CLD will alter the MUC5AC/MUC5B ratio in favour of MUC5AC. This is important, as a higher MUC5AC/MUC5B ratio has been observed in pathogenic conditions including asthma [69]. The reason for the more pathogenic nature of MUC5AC is not fully understood. However, the tendency of MUC5AC to form sheets, and increased tethering to the airway epithelium, may play a part in impairing MCC to promote disease [70,71]. Impairing MCC would also be of benefit to fungal species trying to colonise the airway, giving an evolutionary advantage to those that induce *MUC5AC* expression. Future studies providing a clearer understanding of how proteases regulate the expression of *MUC5B* will be important not only in muco-obstructive lung disease, due to its role in MCC [58], but also the wider field of CLD including in idiopathic pulmonary fibrosis where a *MUC5B* promoter polymorphism and impaired MCC are associated with disease development [72,73].

#### 2.3.2. Mucin Secretion

Following translation, mucins are packaged in a dehydrated form in secretory granules. Upon exocytosis the mucins are hydrated, absorbing more than 100 times their volume in water and, in the process, expand and acquire the correct viscoelastic properties to allow effective MCC [74]. Secretion of mucins is an incredibly rapid process occurring within a few hundred milliseconds [75]. Additionally, this secretory process is highly inducible, increasing over 1000-fold in response to certain stimuli [76,77]. Mucus hypersecretion is a major component of muco-obstructive lung diseases associated with declining lung function [78,79]. Metalloproteases including ADAM-10, meprin-α, and MMP-9, as well as the neutrophil serine proteases NE, cathepsin G and proteinase 3, are potent mucus secretagogues, inducing goblet cell degranulation and secretion of mucins from airway submucosal glands [80,81,82,83]. The specific mechanisms through which proteases induce mucin secretion are not fully understood. A number of key pathways have been highlighted in the literature. A study by Takeyama et al. demonstrated that cell-bound NE, but not free NE, could induce goblet cell degranulation, suggesting that a secondary signal may be required from the intercellular adhesion molecule (ICAM)-1 on the neutrophil cell surface to induce degranulation [84]. The intracellular signalling pathways that may be involved in this process were not elucidated in this study. More recently, NE was shown to induce mucin secretion via a protein kinase C (PKC)-dependent mechanism involving phosphorylation of myristoylated alanine-rich C kinase substrate (MARCKS), a PKC target and key regulator of mucin secretion [85]. Additionally, miR-146a negatively regulates NE-induced MUC5AC secretion from AECs through the inactivation of c-Jun N-terminal kinase (JNK) and nuclear factor kappa B (NF-κB) signaling [86]. Much like mucin expression, it is not only human proteases that regulate mucin secretion. Bacterial proteases including *Pseudomonas* elastase B, alkaline protease, and protease IV have all been shown to induce mucin secretion [87].

#### 2.3.3. Mucus Viscoelastic Properties

**Figure 1:**
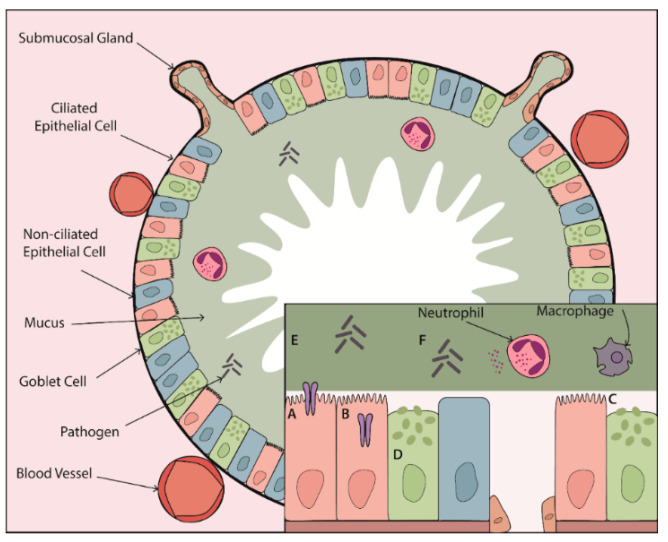
The effect of proteases on mucus and mucociliary clearance in the chronically inflamed airway. Proteases contribute to CLD pathogenesis through their impact on every step of the MCC mechanism. Elevated protease activity leads to (**A**) activation of ENaC and (**B**) loss of CFTR at the epithelial surface contributing to airway surface dehydration. (**C**) Protease-dependent damage to ciliated epithelial cells and cleavage of ciliary proteins leads to ineffective mucus clearance. This clearance defect is compounded by (**D**) protease-mediated increases in mucin expression and secretion from goblet cells and submucosal glands resulting in a highly viscous mucus layer that can no longer be cleared effectively. (**E**) Proteases can degrade mucins and (**F**) induce release of NETs, which may further alter mucus viscoelastic properties. Together, protease-dependent mucin/mucus hypersecretion and mucus dehydration produce highly viscous mucus, setting the stage for mucus plugging in the airways of patients with muco-obstructive lung disease.

Once secreted, gel-forming mucins MUC5AC and MUC5B form part of the mucus gel layer. The concentration of mucins in this layer contributes to its viscoelastic properties. Healthy mucus contains approximately 3% solids, having the consistency of egg whites [9]. However, in chronic lung disease this can increase to up to 15% solids as a result of airway dehydration coupled with increased mucin expression and hypersecretion [9]. However, it is not only the solid content of mucus that determines its viscoelastic properties; a number of other factors influence mucus viscosity including pH, extracellular deoxyribonucleic acid (DNA) content and the presence of mucin crosslinking, which occurs via the formation of disulphide bonds between mucins during oxidative stress [40,57,88]. Besides regulating mucin expression and secretion, proteases also regulate mucus viscoelastic properties by directly acting on secreted mucin proteins. In vitro studies have demonstrated that serine proteases are capable of degrading mucins [89]. While this would seem to suggest that protease activity may decrease mucus viscosity, this has not been directly measured. Importantly MUC5B is required for MCC and therefore its degradation could in fact hinder airway clearance [58]. Furthermore, proteases regulate the release of neutrophil extracellular traps (NETs) [90]. Induction of NET formation and subsequent increases in extracellular DNA may contribute to increased mucus viscosity. NETs also provide a protective lattice around proteases preventing access and inhibition by their natural inhibitors [91,92]. Bacterial species in the airway use mucolytic proteases to promote colonisation by inhibiting entrapment in the mucus layer and to gain access to the airway epithelium. *P. aeruginosa*-derived elastase B (pseudolysin) degrades both MUC5AC and MUC5B [89]. Mucins in the airways are highly sulphated, a mechanism to protect against degradation from bacterial proteases. However, *P. aeruginosa* has evolved the ability to secrete sulfatases, allowing it to bypass this protective barrier [93]. Fungal species including *A. fumigatus* break down mucins, not only to promote colonisation, but also to utilise it as a nutrient source [94]. A summary of the effects of proteases on mucus and MCC in muco-obstructive lung disease can be found in Figure 1.

## 3. Proteases and Mucosal Immunity

### 3.1. Mucus and Mucosal Immunity

The protease-mediated changes in mucus and MCC that occur in CLD do not occur in a vacuum. Indeed, pathogenic changes in mucin expression, secretion, mucus composition and the mucociliary apparatus itself have profound implications for mucosal immunity and inflammation. This is due to increased pathogenic colonisation of the airways, but also because of ‘sterile’ inflammation, caused by hypoxic epithelial cell necrosis within and around mucus plugs, which may contribute to neutrophilic airway inflammation via the release of IL-1α from necrotic cells [95]. The proinflammatory nature of mucus obstruction in the lungs in the absence of bacterial infection has been revealed through studies in pathogen-free ferrets with CF and mice that overexpress the β-subunit of ENaC (βENaC-Tg) with CF-like disease, as well as in children with CF with no detectable bacterial infection [29,96,97,98,99]. In addition, it was shown that excess airway mucus triggers an MMP-12 producing, activated macrophage phenotype in the βENaC-Tg model of muco-obstructive lung disease [100]. Thus, mucus plugging *per se* can trigger airway inflammation and it is well established that muco-obstructive lung conditions feature expanded and phenotypically different cellular populations at the mucosal surface. In this section we will introduce the key contributors to airways mucosal immunity and how proteases affect their recruitment and function in muco-obstructive lung diseases.

Epithelial cells line the airways and present the basic cellular defensive barrier responsible for controlling the movement of host and external factors into and out of the airway lumen. They also sense pathogens and toxins through an array of receptors, and secrete, among others, mucus (as discussed above), antimicrobial peptides (AMPs), and inflammatory mediators [101]. The signals originating from airway epithelial cells, when alive or necrotic, result in the accumulation of immune cells in the airways. Of the immune cells recruited to the muco-obstructed lung, neutrophils and macrophages predominate. Neutrophils are primed and recruited from the vasculature in response to cytokines, chemokines, lipid mediators, damage- and pathogen-associated molecular patterns, growth factors, and activated endothelial cells [102]. A large pool of neutrophils is present in the lung vasculature, facilitating exceedingly rapid transition into the airways. Recruited neutrophils phagocytose pathogens and release a barrage of cytotoxic products including proteases and reactive oxygen species, which may be complexed in NETs. Neutrophilia is common to most CLDs but studies examining the functionality of neutrophils from patients with CLDs have demonstrated that, despite being present in high numbers, neutrophil function is impaired or defective [103,104,105]. 

Lung-resident macrophages can be classified as alveolar macrophages or interstitial macrophages and are phenotypically diverse and highly plastic. They perform a wide variety of functions including the effusive production of a wide range of signalling molecules, efferocytosis, phagocytosis and antigen presentation [106]. The signalling molecules they release also stimulate the release of bone marrow monocytes, which migrate, differentiate, and supplement the resident macrophage population [107]. As with neutrophils, macrophages from patients with CLDs display impaired function, preventing the efficient clearance of pathogens and recruited neutrophils [108,109]. Most protease research in the lung to date has centred on these particular cellular players and they are thought to contribute most significantly to the protease burden in the inflamed lung. However, other myeloid cells, including dendritic cells and eosinophils, and a collection of lymphoid cells, including innate lymphoid cells (ILCs) and so-called unconventional T cells are also present to varying degrees in CLD, and may contribute to protease-mediated disease development, though their roles remain less well defined [110,111,112,113]. Indeed, understanding the protease repertoire of these more uncommon cells, and the impact of proteases on these cells, represents an important area for future work.

### 3.2. Proteases as Regulators of Host Defence

One of the principal roles of these cellular players in the airway is to identify, slow, trap and destroy potentially dangerous stimuli. It was long assumed that upon deployment into the extracellular environment, proteases, and especially the neutrophil serine proteases (NSPs), acted as important effectors of microbial killing. However, it appears that this is not necessarily the case [114,115] and that the primary antimicrobial abilities of the NSPs are against phagocytosed pathogens within the phagocytic vacuole [116], where NSP concentrations are much higher than in the extracellular environment during degranulation. Indeed, at high concentrations, NSPs have demonstrated potent antimicrobial activity against many respiratory pathogens including *Streptococcus pneumoniae*, *Klebsiella pneumoniae* and *P. aeruginosa* [117,118,119]. In macrophages, the mantle for intracellular pathogen killing by non-oxidative means is also taken up by the lysosomal cathepsins [120,121]. Furthermore, the processing and trafficking of certain Toll-like receptors (TLRs), vital receptors for the recognition of microbial membrane products and nucleic acids, appears to be dependent on intracellular cathepsins and asparagine endopeptidase, also known as legumain [122,123]. In macrophages and dendritic cells, cathepsins also assist the development of adaptive immunity by generating antigenic peptides from lysosomally-degraded pathogens for presentation to adaptive immune cells [124]. 

The airways in patients with CLDs are rich in proinflammatory signals, as the immune system attempts to manage pathogenic insults. As part of the multi-layered control of inflammatory signalling that the immune system employs, numerous proteases are known to participate in the processing of cytokine and chemokines, modulating their function. Indeed, it has been proposed that NSPs secreted following microbe phagocytosis are actually more disposed towards escalating inflammatory responses by this method than extracellular microbicidal activity [115]. This is thought to be mediated through the cleavage of interleukin-1 (IL-1) family cytokines, generating an array of interleukins with modified chemotactic abilities. Serine, cysteine and MMPs, all of which are present in the chronically inflamed airway, share this ability and modify, among others, IL-8, CXCL5, CCL15 and chemerin [125,126,127,128,129]. Growth factors and cytokines can also be liberated by proteases from the extracellular matrix (ECM) of the lung and cellular membranes through ‘sheddase’ activity. This is a well-known ability of MMPs and ADAM proteases, and contributes to the release of soluble tumour necrosis factor (TNF), TNFR and IL-6R [130], as well as chemoattractant matrikine fragments like proline-glycine-proline, which can signal via CXCR1 and CXCR2 [131,132]. Extracellular proteases contribute substantially to ECM cleavage and airway remodelling, in both normal and pathological settings, though a thorough investigation of these aspects of protease function is beyond the scope of this review. For further reading, the reader is directed towards the following reviews [131,133,134]. Overall, proteases are important effectors of pathogen removal and act as intermediaries in the escalation of appropriate immune responses to pathogenic stimuli in the airways.

Despite these abilities to facilitate and positively escalate the host response during inflammation, dysregulated protease activity has also been shown to compromise host defence. Numerous studies have demonstrated the ability of proteases to cleave AMPs like lactoferrin, LL-37 and defensins, while also liberating iron from ferritin, providing nutrients for bacterial outgrowth [135,136,137]. Surfactant proteins are also readily degraded by host and bacterial proteases, compromising their pathogen-opsonising and direct antimicrobial functions [138,139,140,141]. 

Respiratory viruses are thought to play an important part in the exacerbations that punctuate the progressive decline of lung function in patients with CLD [142] and certain respiratory viruses use host proteases to their advantage during infection. For example, the influenza virus haemagglutinin precursor is cleaved by membrane-bound respiratory trypsin-like serine proteases to its fusion-active form, allowing entry into, and spread from, airway epithelial cells [143]. Likewise, human coronaviruses, including the severe acute respiratory syndrome coronaviruses, appear to use host-derived serine and/or cysteine proteases to prime the spike glycoprotein to facilitate viral invasion [144]. Thus, proteases appear to both assist the pathogenic side of the airways’ arms race and impede the armouring of the host side.

### 3.3. Self-Perpetuating Protease Activity

Though proteases are generally well-regulated during health, a pro-proteolytic environment at the mucosal surface can be propagated by proteases, using at least two mechanisms: degradation of endogenous protease inhibitors and activation of other proteases. The major antiproteases for each class of protease are present at the airway surface and exhibit protease inhibitory and host defence abilities. For example, secretory leukocyte protease inhibitor (SLPI), elafin and α_1_-antitrypsin (A1-AT) inhibit NSPs but also variously disrupt bacterial membranes by high cationicity, bind and neutralise bacteria, and demonstrate potent anti-inflammatory effects independent of protease inhibition [4,145,146]. Similar anti-inflammatory effects have been reported for cysteine protease inhibitors (cystatins), while tissue inhibitors of metalloproteinases (TIMPs) seem to possess some cytokine-like signalling abilities [147,148]. Elevated levels of degraded antiproteases are present in lung fluids from patients with CLD, intimating that proteases may participate in their own deregulation during disease. The cleavage of antiproteases that inhibit serine proteases is the best studied and in different disease settings has been reported to be caused by NE, extracellular proteasome, cathepsins B, L and S and MMP-9 [149,150,151,152,153,154,155]. NE also degrades TIMP-1 and cystatin C [155,156]. Thus, in the inflamed lung, it is important to view increased levels of proteases in the context of a diminished antiprotease shield, accentuating the imbalance. It is also worth noting that, as has been mentioned, some proteases, including NE, cathepsin G and MMP-12, are not exclusively present as free soluble forms [100,157,158], and as such, can be shielded from their inhibitors by remaining membrane-associated or complexed with extracellular molecules [91,159]. Shielded protease localisations are increasingly being recognised in CLDs and assessing these inaccessible forms by, for example, fluorescence resonance energy transfer (FRET)-based assays [160,161], may bring to light a more substantial protease burden than has been appreciated to date, as well as providing more effective targeting strategies for pharmacological inhibitors.

Another in-built mechanism to prevent aberrant proteolysis is the synthesis of proteases as inactive precursors that must be cleaved to become active. However, as with antiproteases, inhibitory domains are readily degraded in an already protease-rich environment, allowing protease activity to stimulate more protease activity. This protease cascade is illustrated elegantly in the multistep maturation of cathepsin C, the master regulator of the NSPs, by cysteine cathepsins [162]. Once this is completed, activated cathepsin C is then able to process NE [163], which goes on to facilitate the maturation of MMP-9 [155,164]. Thus, with this and other protease cascades, there is scope for rapid and uncontrolled expansion of protease activity in the chronically inflamed lung.

### 3.4. Protease Signalling and Epithelial Integrity

Many reports have highlighted that proteases contribute to the recruitment of immune cells to the airway and, as has been mentioned, this may be explained in part by the activation of cytokines and chemokines. However, proteases also directly affect cellular inflammatory pathways through PARs expressed on epithelial, endothelial, and immune cells. PARs are comprised of an *N*-terminal ligand tethered to a seven transmembrane domain G protein-coupled receptor. The tethered ligand can be cleaved at discrete sites by proteases and subsequently binds to the receptor, initiating various signalling pathways [165]. Salient roles in pulmonary disease have been demonstrated for PAR1 and PAR2, though the relevance of PAR3 and PAR4 in this context has yet to be established. PAR1 can be activated by a plethora of common CLD proteases including the NSPs and several MMPs, while PAR2 is also activated by cathepsin S; both PAR1 and PAR2 are activated by proteases of the coagulation cascade, such as thrombin, and the full gamut of proteases responsible for PAR activation is not yet clear. An additional layer of complexity is added to PAR signalling by the fact that some proteases ‘alternatively activate’ PARs, which can produce different downstream signals, or render the receptor unresponsive to other proteases. To date, pulmonary PAR research has focussed on acute lung injury and fibrosis [166,167,168]. In these contexts, PAR activation induces the release of potent inflammatory cytokines and chemokines such as CCL2, IL-6 and TNF-α, and can also stimulate the upregulation of inflammatory cell adhesion molecules like P-selectin and ICAM-1 [169,170]. However, there is growing evidence of PAR-related chronic lung damage. In vivo blockade of PAR2 reduces pulmonary inflammation in the βENaC-Tg mouse model, an effect that may be the result of diminished CTSS-PAR2 signalling [171]. MMP-12 is able to upregulate early growth response factor 1 (Egr1) and placental growth factor (PGF) through activation of PAR1 in bronchial epithelial cells and in vivo, triggering epithelial apoptosis [172]. NE has also been shown to mediate epithelial apoptosis in a similar manner [173]. PAR signalling is dynamic and is shaped by the proteases present and possibly the concentrations at which they are present [174]. Therefore, the PAR-related pathways that are activated in the lungs are likely to vary between CLDs and stages of disease, depending on proteolytic burden.

An additional way in which proteases may alter mucosal immunity is by weakening the confluent epithelial cell structure of the airways. Epithelial barrier function relies on tight junctions composed of claudins and occludins, which are selectively permeable to allow the controlled movement of water and solutes to the exclusion of high molecular weight proteins and oedema fluid [175]. Some proteases, and especially the meprin metalloproteases, cleave tight junction proteins, potentially aiding the transmigration of immune cells across the epithelial barrier [176,177]. However, it is not clear whether proteases contribute significantly to the loss of tight junction integrity in the context of CLD or if changes in barrier function are mostly a response to inflammatory signals and fluid balance across the epithelium [178].

## 4. Targeting Proteases in Muco-Obstructive Lung Disease

The use of recombinant or small molecule protease inhibitors as therapeutics in CLD is not widely practiced. In spite of protease inhibitors demonstrating efficacy in pre-clinical in vivo models of muco-obstructive lung disease, the translation of these in vivo studies into the clinic has been disappointing [100,171,179,180,181]. With the exception of A1-AT augmentation therapy, no protease inhibitors have been approved for clinical use in treating CLD [182,183,184]. Protease inhibitors that have been tested in clinical trials against muco-obstructive lung diseases are included in Table 1. There could be a number of reasons for the observed poor clinical efficacy. As previously mentioned, the presence of membrane-bound proteases in the chronically inflamed airways may limit antiprotease efficacy. A number of studies have shown that cell surface or exosome-bound proteases are resistant to inhibition [159,185,186]. Additionally, functional redundancy between proteases in a subfamily means that inhibiting a single protease target may not always be appropriate [187]. However, inhibition of whole protease subfamilies results in unwanted side effects, as has been observed with broad spectrum-MMP inhibitors [188]. The use of inhibitors that specifically target extracellular protease activity could mitigate undesirable effects associated with intracellular protease inhibition.

Determining the role of specific proteases in particular pathways of interest will be key, though the complex interplay between proteases in the chronically inflamed airways makes this a difficult task. Interestingly, even single protease inhibition strategies have effects on the wider protease web. For instance, NE has been shown to regulate the expression of cysteine proteases (CTSB) and MMPs (MMP-2) in a murine *Pseudomonas* infection model and in human macrophages [209]. In subsequent studies, NE inhibition by A1-AT rescued this effect, diminishing cathepsin and MMP-mediated cleavage of AMPs [210]. Understanding such protease hierarchies (including those mentioned in Section 3.3) has important implications for choosing specific proteases to target: can the bulk of the protease burden be efficiently reduced by targeting a single, or very few proteases? Can we identify beforehand which proteases will be impacted by an inhibitor therapy, and hence, predict potential side effects?

Additionally, careful consideration of trial design will also be important, including study length and outcomes. Short-term changes in inflammation and lung function may be less relevant than the long-term impacts of protease inhibition. The ability of protease inhibitors to improve MCC in clinical trials has not yet been examined. With preclinical data demonstrating a role for proteases in multiple stages of the MCC mechanism, they represent an interesting alternative to currently available mucoactive drugs. Mucolytic drugs, such as dornase alfa and hypertonic saline have demonstrated efficacy in CF, however, studies in other muco-obstructive lung conditions including COPD and non-CF bronchiectasis have shown mixed results [211,212,213,214,215]. Heterogeneity in these conditions may be an issue; stratification or recruitment of specific disease sub-phenotypes that suffer from chronic cough and sputum production may help to uncover efficacy in these conditions. Mucoactive drugs may provide a benefit beyond their own direct efficacy. An ever-present challenge in muco-obstructive lung disease is delivery of a drug to a target that is surrounded by a complex mucus barrier [216]. As such, mucolytics given in tandem with other treatments could improve delivery of the drug to its target, potentially bolstering effects. In this context, clinical studies undertaking combination therapies with protease inhibitors and mucoactive drugs may represent an interesting future direction.

## 5. Conclusions

The pathogenesis of muco-obstructive lung diseases is complex and is heavily influenced by mucus dynamics, host defence mechanisms and inflammatory responses in the airways. In this review we have discussed the involvement of proteases in controlling these factors and highlighted the importance and therapeutic potential of limiting excessive proteolytic activity in the airways. Considering the limited therapeutic options available for patients with progressive and, as yet irreversible chronic lung diseases like COPD, CF and non-CF bronchiectasis, therapies that target proteases or protease-mediated pathways may have a valuable future as adjuncts to current approaches.

## Figures and Tables

**Table 1:** Overview of clinical trials undertaken using protease inhibitors in patients with muco-obstructive lung diseases including CF, COPD, and bronchiectasis.

Target Protease	Inhibitor	Disease	Stage	Outcome
NE	A1-AT	CF	II	Reduced inflammation, no effect on lung function [189,190]
		Bronchiectasis	I	Results unpublished [191]
		COPD/A1-AT deficiency	II/III	Reduced serine protease levels, reduces elastin degradation in the lung, reduced inflammation [192,193,194]
	AZD9668	CF	II	Reduced inflammation, no effect on lung function [195]
		COPD	II	No changes in lung function or inflammation [196,197]
		Bronchiectasis	II	Improved lung function and reduced sputum inflammatory biomarkers [198]
	AZD6553	COPD	I	Terminated due to emerging PK profile that could not be aligned to the known pharmaceutical properties of the IMP [199]
	Alvelestat	COPD	II	Currently recruiting [200]
	POL6014	CF	I	No serious adverse effects noted [201,202]
	CHF6333	CF + non-CF bronchiectasis	I	Results unpublished [203]
	BI 1323495	Bronchiectasis	I	Currently recruiting [204]
	BAY85-8501	Bronchiectasis	II	No changes in lung function or inflammation [205,206]
MMP-9/-12	AZD1236	COPD	II	No clinical efficacy observed [207]
Cathepsin C	Brensocatib	Bronchiectasis	II	Improved clinical outcomes with reduced NE activity, reduced time to first exacerbation [208]

## Abbreviations

A1-AT	α_1_-antitrypsin
ADAM	A disintegrin and a metalloprotease
AEC	Airway epithelial cell
AMP	Antimicrobial peptide
ASL	Airway surface liquid
βENaC-Tg	Transgenic mouse overexpressing the β-subunit of epithelial sodium channel
cAMP	Cyclic adenosine monophosphate
CAP	Channel-activating protease
CBF	Ciliary beat frequency
CF	Cystic fibrosis
CFTR	Cystic fibrosis transmembrane conductance regulator
CLD	Chronic lung disease
COPD	Chronic obstructive pulmonary disease
CTSB	Cathepsin B
CTSS	Cathepsin S
DNA	Deoxyribonucleic acid
ECM	Extracellular matrix
EGFR	Epidermal growth factor receptor
Egr	Early growth response
ENaC	Epithelial sodium channel
ERK	Extracellular receptor kinase
FRET	Fluorescence resonance energy transfer
HAT	Human airway trypsin-like protease
ICAM	Intercellular adhesion molecule
IMP	Investigational medical product
JNK	c-Jun N-terminal kinase
MARCKS	Myristoylated alanine-rich C kinase substrate
MCC	Mucociliary clearance
MMP	Matrix metalloprotease
mRNA	Messenger ribonucleic acid
NE	Neutrophil elastase
NET	Neutrophil extracellular trap
NF-κB	Nuclear factor κB
NSP	Neutrophil serine protease
PAR	Protease-activated receptor
PCD	Primary ciliary dyskinesia
PK	Pharmacokinetic
PKC	Protein kinase C
PGF	Placental growth factor
SLPI	Secretory leukocyte protease inhibitor
TIMP	Tissue inhibitor of metalloproteinases
TLR	Toll-like receptor
TMPRSS	Transmembrane protease, serine
TNF	Tumour necrosis factor

## References

[1] Kappelhoff R., Puente X.S., Wilson C.H., Seth A., López-Otín C., Overall C.M. (2017). Overview of transcriptomic analysis of all human proteases, non-proteolytic homologs and inhibitors: Organ, tissue and ovarian cancer cell line expression profiling of the human protease degradome by the CLIP-CHIPTM DNA microarray. BBA-Mol. Cell Res..

[2] Taggart C., Mall M.A., Lalmanach G., Cataldo D., Ludwig A., Janciauskiene S., Heath N., Meiners S., Overall C.M., Schultz C. (2017). Protean proteases: At the cutting edge of lung diseases. Eur. Respir. J..

[3] Taggart C.C., Greene C.M., Carroll T.P., O’Neill S.J., McElvaney N.G. (2005). Elastolytic proteases: Inflammation resolution and dysregu-lation in chronic infective lung disease. Am. J. Respir. Crit. Care Med..

[4] Greene C.M., McElvaney N.G. (2009). Proteases and antiproteases in chronic neutrophilic lung disease-relevance to drug discovery. Br. J. Pharmacol..

[5] McKelvey M.C., Weldon S., McAuley D.F., Mall M.A., Taggart C.C. (2020). Targeting Proteases in Cystic Fibrosis Lung Disease. Paradigms, Progress, and Potential. Am. J. Respir. Crit. Care Med..

[6] Knowles M.R., Boucher R.C. (2002). Mucus clearance as a primary innate defense mechanism for mammalian airways. J. Clin. Investig..

[7] Mall M.A. (2008). Role of Cilia, Mucus, and Airway Surface Liquid in Mucociliary Dysfunction: Lessons from Mouse Models. J. Aerosol Med. Pulm. Drug Deliv..

[8] Bustamante-Marin X.M., Ostrowski L.E. (2017). Cilia and Mucociliary Clearance. Cold Spring Harb. Perspect. Biol..

[9] Fahy J.V., Dickey B.F. (2010). Airway Mucus Function and Dysfunction. N. Engl. J. Med..

[10] Boucher R.C. (2019). Muco-Obstructive Lung Diseases. N. Engl. J. Med..

[11] Anderson W.H., Coakley R.D., Button B., Henderson A.G., Zeman K.L., Alexis N.E., Peden D.B., Lazarowski E.R., Davis C.W., Bailey S. (2015). The Relationship of Mucus Concentration (Hydration) to Mucus Osmotic Pressure and Transport in Chronic Bronchitis. Am. J. Respir. Crit. Care Med..

[12] Button B., Anderson W.H., Boucher R.C. (2016). Mucus Hyperconcentration as a Unifying Aspect of the Chronic Bronchitic Phenotype. Ann. Am. Thorac. Soc..

[13] Henderson A.G., Ehre C., Button B., Abdullah L.H., Cai L.-H., Leigh M.W., DeMaria G.C., Matsui H., Donaldson S.H., Davis C.W. (2014). Cystic fibrosis airway secretions exhibit mucin hyperconcentration and increased osmotic pressure. J. Clin. Investig..

[14] Tarran R. (2004). Regulation of Airway Surface Liquid Volume and Mucus Transport by Active Ion Transport. Proc. Am. Thorac. Soc..

[15] Tarran R., Button B., Picher M., Paradiso A.M., Ribeiro C.M., Lazarowski E.R., Zhang L., Collins P.L., Pickles R.J., Fredberg J.J. (2005). Normal and cystic fibrosis airway surface liquid homeostasis: The effects of phasic shear stress and viral infections. J. Biol. Chem..

[16] Kunzelmann K., Greger R. (1995). Na^+^ and Cl^−^ conductances in airway epithelial cells: Increased Na^+^ conductance in cystic fibrosis. Pflügers Archiv-Eur. J. Physiol..

[17] Riordan J.R., Rommens J.M., Kerem B., Alon N., Rozmahel R., Grzelczak Z., Zielenski J., Lok S., Plavsic N., Chou J.L. (1989). Identification of the cystic fibrosis gene: Cloning and characterization of complementary DNA. Science.

[18] Hughey R.P., Bruns J.B., Kinlough C.L., Harkleroad K.L., Tong Q., Carattino M.D., Johnson J.P., Stockand J.D., Kleyman T.R. (2004). Epithelial Sodium Channels Are Activated by Furin-dependent Proteolysis. J. Biol. Chem..

[19] Bruns J.B., Carattino M.D., Sheng S., Maarouf A.B., Weisz O.A., Pilewski J.M., Hughey R.P., Kleyman T.R. (2007). Epithelial Na^+^ Channels Are Fully Activated by Furin- and Prostasin-dependent Release of an Inhibitory Peptide from the γ-Subunit. J. Biol. Chem..

[20] Haerteis S., Krappitz M., Bertog M., Krappitz A., Baraznenok V., Henderson I., Lindström E., Murphy J.E., Bunnett N.W., Korbmacher C. (2012). Proteolytic activation of the epithelial sodium channel (ENaC) by the cysteine protease cathepsin-S. Pflügers Archiv-Eur. J. Physiol..

[21] García-Caballero A., Dang Y., He H., Stutts M.J. (2008). ENaC Proteolytic Regulation by Channel-activating Protease 2. J. Gen. Physiol..

[22] Caldwell R.A., Boucher R.C., Stutts M.J. (2004). Serine protease activation of near-silent epithelial Na+ channels. Am. J. Physiol. Physiol..

[23] Larionov A., Dahlke E., Kunke M., Rodriguez L.Z., Schiessl I.M., Magnin J., Kern U., Alli A.A., Mollet G., Schilling O. (2019). Cathepsin B increases ENaC activity leading to hypertension early in nephrotic syndrome. J. Cell. Mol. Med..

[24] Caldwell R.A., Boucher R.C., Stutts M.J. (2005). Neutrophil elastase activates near-silent epithelial Na^+^ channels and increases airway epithelial Na^+^ transport. Am. J. Physiol. Cell. Mol. Physiol..

[25] Shuto T., Kamei S., Nohara H., Fujikawa H., Tasaki Y., Sugahara T., Ono T., Matsumoto C., Sakaguchi Y., Maruta K. (2016). Pharmacological and genetic reappraisals of protease and oxidative stress pathways in a mouse model of obstructive lung diseases. Sci. Rep..

[26] Butterworth M.B., Zhang L., Liu X., Shanks R.M., Thibodeau P.H. (2014). Modulation of the Epithelial Sodium Channel (ENaC) by Bacterial Metalloproteases and Protease Inhibitors. PLoS ONE.

[27] Mall M., Grubb B.R., Harkema J.R., O’Neal W.K., Boucher R.C., Neal W.K.O. (2004). Increased airway epithelial Na^+^ absorption produces cystic fibrosis-like lung disease in mice. Nat. Med..

[28] Zhao R., Liang X., Zhao M., Liu S.-L., Huang Y., Idell S., Li X., Ji H.-L. (2014). Correlation of Apical Fluid-Regulating Channel Proteins with Lung Function in Human COPD Lungs. PLoS ONE.

[29] Mall M.A., Harkema J.R., Trojanek J.B., Treis D., Livraghi A., Schubert S., Zhou Z., Kreda S.M., Tilley S.L., Hudson E.J. (2008). Development of chronic bronchitis and emphysema in β-epithelial Na+ channel-overexpressing mice. Am. J. Respir. Crit. Care Med..

[30] Kerem E., Bistritzer T., Hanukoglu A., Hofmann T., Zhou Z., Bennett W., MacLaughlin E., Barker P., Nash M., Quittell L. (1999). Pulmonary Epithelial Sodium-Channel Dysfunction and Excess Airway Liquid in Pseudohypoaldosteronism. N. Engl. J. Med..

[31] Averna M., Stifanese R., Grosso R., Pedrazzi M., De Tullio R., Salamino F., Pontremoli S., Melloni E. (2010). Role of calpain in the regulation of CFTR (cystic fibrosis transmembrane conductance regulator) turnover. Biochem. J..

[32] Matos A.M., Pinto F.R., Barros P., Amaral M.D., Pepperkok R., Matos P. (2019). Inhibition of calpain 1 restores plasma membrane stability to pharmacologically rescued Phe508del-CFTR variant. J. Biol. Chem..

[33] Averna M., Stifanese R., De Tullio R., Minicucci L., Cresta F., Palena S., Salamino F., Pontremoli S., Melloni E. (2011). Evidence for alteration of calpain/calpastatin system in PBMC of cystic fibrosis patients. Biochim. Biophys. Acta Mol. Basis Dis..

[34] Averna M., Pedrazzi M., Minicucci L., De Tullio R., Cresta F., Salamino F., Pontremoli S., Melloni E. (2013). Calpain Inhibition Promotes the Rescue of F508del-CFTR in PBMC from Cystic Fibrosis Patients. PLoS ONE.

[35] Le Gars M., Descamps D., Roussel D., Saussereau E., Guillot L., Ruffin M., Tabary O., Hong S.S., Boulanger P., Paulais M. (2013). Neutrophil elastase degrades cystic fibrosis transmembrane conductance regulator via calpains and disables channel function in vitro and in vivo. Am. J. Respir. Crit. Care Med..

[36] Mall M.A., Hartl D. (2014). CFTR: Cystic fibrosis and beyond. Eur. Respir. J..

[37] Bodas M., Min T., Mazur S., Vij N. (2011). Critical Modifier Role of Membrane-Cystic Fibrosis Transmembrane Conductance Regula-tor-Dependent Ceramide Signaling in Lung Injury and Emphysema. J. Immunol..

[38] Cantin A.M., Hanrahan J.W., Bilodeau G., Ellis L., Dupuis A., Liao J., Zielenski J., Durie P. (2006). Cystic Fibrosis Transmembrane Conductance Regulator Function Is Suppressed in Cigarette Smokers. Am. J. Respir. Crit. Care Med..

[39] Clunes L.A., Davies C.M., Coakley R.D., Aleksandrov A.A., Henderson A.G., Zeman K.L., Worthington E.N., Gentzsch M., Kreda S.M., Cholon D. (2011). Cigarette smoke exposure induces CFTR internalization and insolubility, leading to airway surface liquid dehydration. FASEB J..

[40] Pezzulo A.A., Tang X.X., Hoegger M.J., Abou Alaiwa M.H., Ramachandran S., Moninger T.O., Karp P.H., Wohlford-Lenane C.L., Haagsman H.P.v.E.M., Bánfi B. (2012). Reduced airway surface pH impairs bacterial killing in the porcine cystic fibrosis lung. Nature.

[41] Haitchi H.M., Yoshisue H., Ribbene A., Wilson S.J., Holloway J.W., Bucchieri F., Hanley N.A., Wilson D.I., Zummo G., Holgate S.T. (2009). Chronological expression of Ciliated Bronchial Epithelium 1 during pulmonary development. Eur. Respir. J..

[42] Davis C.W., Dickey B.F. (2008). Regulated Airway Goblet Cell Mucin Secretion. Annu. Rev. Physiol..

[43] Lucas J.S., Burgess A., Mitchison H.M., Moya E., Williamson M., Hogg C., on behalf of the National PCD Service, UK (2014). Diagnosis and management of primary ciliary dyskinesia. Arch. Dis. Child..

[44] Wyatt T.A., Spurzem J.R., May K., Sisson J.H. (1998). Regulation of ciliary beat frequency by both PKA and PKG in bovine airway epithelial cells. Am. J. Physiol. Cell. Mol. Physiol..

[45] Di Benedetto G., Magnus C.J., Gray P.T., Mehta A. (1991). Calcium regulation of ciliary beat frequency in human respiratory epithelium in vitro. J. Physiol..

[46] Sutto Z., Conner G.E., Salathe M. (2004). Regulation of human airway ciliary beat frequency by intracellular pH. J. Physiol..

[47] Satir P., Heuser T., Sale W.S. (2014). A Structural Basis for How Motile Cilia Beat. Bioscience.

[48] Hingley S.T., Hastie A.T., Kueppers F., Higgins M.L. (1986). Disruption of respiratory cilia by proteases including those of Pseudomonas aeruginosa. Infect. Immun..

[49] Amitani R., Wilson R., Rutman A., Read R., Ward C., Burnett D., Stockley R.A., Cole P.J. (1991). Effects of Human Neutrophil Elastase andPseudomonas aeruginosaProteinases on Human Respiratory Epithelium. Am. J. Respir. Cell Mol. Biol..

[50] McMahon D.B., Workman A.D., Kohanski M.A., Carey R.M., Freund J.R., Hariri B.M., Chen B., Doghramji L.J., Adappa N.D., Palmer J.N. (2018). Protease-activated receptor 2 activates airway apical membrane chloride permeability and increases ciliary beating. FASEB J..

[51] Gomperts B.N., Gong-Cooper X., Hackett B.P. (2004). Foxj1 regulates basal body anchoring to the cytoskeleton of ciliated pulmonary epithelial cells. J. Cell Sci..

[52] Roberts R.E., Martin M., Marion S., Elumalai G.L., Lewis K., Hallett M.B. (2020). Ca^2+^-activated cleavage of ezrin visualised dynamically in living myeloid cells during cell surface area expansion. J. Cell Sci..

[53] Kirkham S., Sheehan J.K., Knight D., Richardson P.S., Thornton D.J. (2002). Heterogeneity of airways mucus: Variations in the amounts and glycoforms of the major oligomeric mucins MUC5AC and MUC5B. Biochem. J..

[54] Hogg J.C., Chu F.S., Tan W.C., Sin D.D., Patel S.A., Pare P.D., Martinez F.J., Rogers R.M., Make B.J., Criner G.J. (2007). Survival after lung volume reduction in chronic obstructive pulmonary disease: Insights from small airway pathology. Am. J. Respir. Crit. Care Med..

[55] Hill D.B., Long R.F., Kissner W.J., Atieh E., Garbarine I.C., Markovetz M.R., Fontana N.C., Christy M., Habibpour M., Tarran R. (2018). Pathological mucus and impaired mucus clearance in cystic fibrosis patients result from increased concentration, not altered pH. Eur. Respir. J..

[56] Ramsey K.A., Chen A.C.H., Radicioni G., Lourie R., Martin M., Broomfield A., Sheng Y.H., Hasnain S.Z., Radford-Smith G., Simms L.A. (2020). Airway Mucus Hyperconcentration in Non–Cystic Fibrosis Bronchiectasis. Am. J. Respir. Crit. Care Med..

[57] Yuan S., Hollinger M., Lachowicz-Scroggins M.E., Kerr S.C., Dunican E.M., Daniel B.M., Ghosh S., Erzurum S.C., Willard B., Hazen S.L. (2015). Oxidation increases mucin polymer cross-links to stiffen airway mucus gels. Sci. Transl. Med..

[58] Roy M.G., Livraghi-Butrico A., Fletcher A.A., McElwee M.M., Evans S.E., Boerner R.M., Alexander S.N., Bellinghausen L.K., Song A.S., Petrova Y.M. (2014). Muc5b is required for airway defence. Nat. Cell Biol..

[59] Ja S.K., Kim Y.D., Jetten A.M., Belloni P., Nettesheim P. (2002). Overexpression of mucin genes induced by interleukin-1β, tumor necrosis factor-α, lipopolysaccharide, and neutrophil elastase is inhibited by a retinoic acid receptor α antagonist. Exp. Lung Res..

[60] Voynow J.A., Rosenthal Young L., Wang Y., Horger T., Rose M.C., Fischer B.M. (1999). Neutrophil elastase increases MUC5AC mRNA and protein expression in respiratory epithelial cells. Am. J. Physiol. Lung Cell. Mol. Physiol..

[61] Fischer B., Voynow J. (2000). Neutrophil elastase induces MUC5AC messenger RNA expression by an oxidant-dependent mecha-nism. Chest.

[62] Shao M.X.G., Nadel J.A. (2005). Neutrophil Elastase Induces MUC5AC Mucin Production in Human Airway Epithelial Cells via a Cascade Involving Protein Kinase C, Reactive Oxygen Species, and TNF-α-Converting Enzyme. J. Immunol..

[63] Deshmukh H.S., Case L.M., Wesselkamper S.C., Borchers M.T., Martin L.D., Shertzer H.G., Nadel J.A., Leikauf G.D. (2005). Metalloproteinases Mediate Mucin 5AC Expression by Epidermal Growth Factor Receptor Activation. Am. J. Respir. Crit. Care Med..

[64] Chokki M., Yamamura S., Eguchi H., Masegi T., Horiuchi H., Tanabe H., Kamimura T., Yasuoka S. (2004). Human Airway Trypsin-Like Protease Increases Mucin Gene Expression in Airway Epithelial Cells. Am. J. Respir. Cell Mol. Biol..

[65] Kamei S., Fujikawa H., Nohara H., Ueno-Shuto K., Maruta K., Nakashima R., Kawakami T., Matsumoto C., Sakaguchi Y., Ono T. (2018). Zinc Deficiency via a Splice Switch in Zinc Importer ZIP2/SLC39A2 Causes Cystic Fibrosis-Associated MUC5AC Hypersecretion in Airway Epithelial Cells. EBioMedicine.

[66] Oguma T., Asano K., Tomomatsu K., Kodama M., Fukunaga K., Shiomi T., Ohmori N., Ueda S., Takihara T., Shiraishi Y. (2011). Induction of Mucin and MUC5AC Expression by the Protease Activity of Aspergillus fumigatus in Airway Epithelial Cells. J. Immunol..

[67] Saunders R.V., Modha D.E., Claydon A., Gaillard E.A. (2016). Chronic Aspergillus fumigatus colonization of the pediatric cystic fibrosis airway is common and may be associated with a more rapid decline in lung function. Med. Mycol..

[68] Wu X., Lee B., Zhu L., Ding Z., Chen Y. (2020). Exposure to mold proteases stimulates mucin production in airway epithelial cells through Ras/Raf1/ERK signal pathway. PLoS ONE.

[69] Woodruff P.G., Modrek B., Choy D.F., Jia G., Abbas A.R., Ellwanger A., Arron J.R., Koth L.L., Fahy J.V. (2009). T-helper Type 2–driven Inflammation Defines Major Subphenotypes of Asthma. Am. J. Respir. Crit. Care Med..

[70] Bonser L.R., Zlock L., Finkbeiner W., Erle D.J. (2016). Epithelial tethering of MUC5AC-rich mucus impairs mucociliary transport in asthma. J. Clin. Investig..

[71] Ostedgaard L.S., Moninger T.O., McMenimen J.D., Sawin N.M., Parker C.P., Thornell I.M., Powers L.S., Gansemer N.D., Bouzek D.C., Cook D.P. (2017). Gel-forming mucins form distinct morphologic structures in airways. Proc. Natl. Acad. Sci. USA.

[72] Seibold M.A., Wise A.L., Speer M.C., Steele M.P., Brown K.K., Loyd J.E., Fingerlin T.E., Zhang W., Gudmundsson G., Groshong S.D. (2011). A Common MUC5B Promoter Polymorphism and Pulmonary Fibrosis. N. Engl. J. Med..

[73] Duerr J., Leitz D.H.W., Szczygiel M., Dvornikov D., Fraumann S.G., Kreutz C., Zadora P.K., Agircan A.S., Konietzke P., Engelmann T.A. (2020). Conditional deletion of Nedd4–2 in lung epi-thelial cells causes progressive pulmonary fibrosis in adult mice. Nat. Commun..

[74] Jaramillo A.M., Azzegagh Z., Tuvim M.J., Dickey B.F. (2018). Airway Mucin Secretion. Ann. Am. Thorac. Soc..

[75] Shumilov D., Popov A., Fudala R., Akopova I., Gryczynski I., Borejdo J., Gryczynski Z., Grygorczyk R. (2014). Real-time imaging of exocytotic mucin release and swelling in Calu-3 cells using acridine orange. Methods.

[76] Davis C.W., Dowell M.L., Lethem M., Van Scott M. (1992). Goblet cell degranulation in isolated canine tracheal epithelium: Response to exogenous ATP, ADP, and adenosine. Am. J. Physiol. Cell Physiol..

[77] Lethem M.I., Dowell M.L., Van Scott M., Yankaskas J.R., Egan T., Boucher R.C., Davis C.W. (1993). Nucleotide Regulation of Goblet Cells in Human Airway Epithelial Explants: Normal Exocytosis in Cystic Fibrosis. Am. J. Respir. Cell Mol. Biol..

[78] Vestbo J., Prescott E., Lange P. (1996). Association of chronic mucus hypersecretion with FEV1 decline and chronic obstructive pulmonary disease morbidity. Am. J. Respir. Crit. Care Med..

[79] Martínez-Rivera C., Crespo A., Pinedo-Sierra C., García-Rivero J.L., Pallarés-Sanmartín A., Marina-Malanda N., Pascual-Erquicia S., Padilla A., Mayoralas-Alises S., Plaza V. (2018). Mucus hypersecretion in asthma is associated with rhinosinusitis, polyps and exacerbations. Respir. Med..

[80] Lemjabbar H., Basbaum C. (2002). Platelet-activating factor receptor and ADAM10 mediate responses to Staphylococcus aureus in epithelial cells. Nat. Med..

[81] Nadel J.A. (1991). Role of Mast Cell and Neutrophil Proteases in Airway Secretion. Am. Rev. Respir. Dis..

[82] Lundgren J.D., Rieves R.D., Mullol J., Logun C., Shelhamer J.H. (1994). The effect of neutrophil proteinase enzymes on the release of mucus from feline and human airway cultures. Respir. Med..

[83] Witko-Sarsat V., Halbwachs-Mecarelli L., Schuster A., Nusbaum P., Ueki I., Canteloup S., Lenoir G., Descamps-Latscha B., Nadel J.A. (1999). Proteinase 3, a Potent Secretagogue in Airways, Is Present in Cystic Fibrosis Sputum. Am. J. Respir. Cell Mol. Biol..

[84] Takeyama K., Agustí C., Ueki I., Lausier J., Cardell L.O., Nadel J.A. (1998). Neutrophil-dependent goblet cell degranulation: Role of membrane-bound elastase and adhesion molecules. Am. J. Physiol. Content.

[85] Park J.-A., He F., Martin L.D., Li Y., Chorley B.N., Adler K.B. (2005). Human Neutrophil Elastase Induces Hypersecretion of Mucin from Well-Differentiated Human Bronchial Epithelial Cells in Vitro via a Protein Kinase Cδ-Mediated Mechanism. Am. J. Pathol..

[86] Zhong T., Perelman J.M., Kolosov V.P., Zhou X.-D. (2011). MiR-146a negatively regulates neutrophil elastase-induced MUC5AC secretion from 16HBE human bronchial epithelial cells. Mol. Cell. Biochem..

[87] Klinger J.D., Tandler B., Liedtke C.M., Boat T.F. (1984). Proteinases of Pseudomonas aeruginosa evoke mucin release by tracheal epithe-lium. J. Clin. Investig..

[88] Puchelle E., Zahm J.-M., De Bentzmann S., Grosskopf C., Shak S., Mougel D., Polu J.-M. (1996). Effects of rhDNase on purulent airway secretions in chronic bronchitis. Eur. Respir. J..

[89] Henke M.O., John G., Rheineck C., Chillappagari S., Naehrlich L., Rubin B.K. Serine Proteases Degrade Airway Mucins in Cystic Fibrosis. Proceedings of the American Thoracic Society 2011 International Conference.

[90] Majewski P., Majchrzak-Gorecka M., Grygier B., Skrzeczynska-Moncznik J., Osiecka O., Cichy J. (2016). Inhibitors of Serine Proteases in Regulating the Production and Function of Neutrophil Extracellular Traps. Front. Immunol..

[91] Dubois A.V., Gauthier A., Bréa D., Varaigne F., Diot P., Gauthier F., Attucci S. (2012). Influence of DNA on the Activities and Inhibition of Neutrophil Serine Proteases in Cystic Fibrosis Sputum. Am. J. Respir. Cell Mol. Biol..

[92] Guerra M., Halls V.S., Schatterny J., Hagner M., Mall M.A., Schultz C. (2020). Protease FRET Reporters Targeting Neutrophil Extracellular Traps. J. Am. Chem. Soc..

[93] Robinson C.V., Elkins M.R., Bialkowski K.M., Thornton D.J., Kertesz M.A. (2012). Desulfurization of mucin by Pseudomonas aeruginosa: Influence of sulfate in the lungs of cystic fibrosis patients. J. Med. Microbiol..

[94] Cowley A.C., Thornton D.J., Denning D.W., Horsley A. (2016). Aspergillosis and the role of mucins in cystic fibrosis. Pediatr. Pulmonol..

[95] Seibold M.A., Wise A.L., Speer M.C., Steele M.P., Brown K.K., Loyd J.E., Fingerlin T.E., Zhang W., Gudmundsson G., Groshong S.D. (2015). Hypoxic epithelial necrosis triggers neutrophilic inflammation via IL-1 receptor signaling in cystic fibrosis lung disease. Am. J. Respir. Crit. Care Med..

[96] Rosen B.H., Evans T.I.A., Moll S.R., Gray J.S., Liang B., Sun X., Zhang Y., Jensen-Cody C.W., Swatek A.M., Zhou W. (2018). Infection is not required for mucoinflammatory lung disease in CFTR-Knockout ferrets. Am. J. Respir. Crit. Care Med..

[97] Sly P.D., Brennan S., Gangell C., De Klerk N., Murray C., Mott L., Stick S.M., Robinson P.J., Robertson C.F., Ranganathan S.C. (2009). Lung Disease at Diagnosis in Infants with Cystic Fibrosis Detected by Newborn Screening. Am. J. Respir. Crit. Care Med..

[98] Sly P.D., Gangell C.L., Chen L., Ware R.S., Ranganathan S., Mott L.S., Murray C.P., Stick S.M. (2013). Risk factors for bronchiectasis in children with cystic fibrosis. N. Engl. J. Med..

[99] Zhou-Suckow Z., Duerr J., Hagner M., Mall M.A. (2017). Airway mucus, inflammation and remodeling: Emerging links in the patho-genesis of chronic lung diseases. Cell Tissue Res..

[100] Trojanek J.B., Cobos-Correa A., Diemer S., Kormann M., Schubert S.C., Zhou-Suckow Z., Agrawal R., Duerr J., Wagner C.J., Schatterny J. (2014). Airway Mucus Obstruction Triggers Macrophage Activation and Matrix Metalloproteinase 12–Dependent Emphysema. Am. J. Respir. Cell Mol. Biol..

[101] Hiemstra P.S., McCray P.B., Bals R. (2015). The innate immune function of airway epithelial cells in inflammatory lung disease. Eur. Respir. J..

[102] Jasper A.E., McIver W.J., Sapey E., Walton G.M. (2019). Understanding the role of neutrophils in chronic inflammatory airway disease. F1000Research.

[103] Hoenderdos K., Condliffe A. (2013). The Neutrophil in Chronic Obstructive Pulmonary Disease. Too Little, Too Late or Too Much, Too Soon?. Am. J. Respir. Cell Mol. Biol..

[104] Bedi P., Davidson D.J., McHugh B.J., Rossi A.G., Hill A.T. (2018). Blood Neutrophils Are Reprogrammed in Bronchiectasis. Am. J. Respir. Crit. Care Med..

[105] Laval J., Ralhan A., Hartl D. (2016). Neutrophils in cystic fibrosis. Biol. Chem..

[106] Kapellos T.S., Bassler K., Aschenbrenner A.C., Fujii W., Schultze J.L. (2018). Dysregulated Functions of Lung Macrophage Populations in COPD. J. Immunol. Res..

[107] Hartl D., Tirouvanziam R., Laval J., Greene C.M., Habiel D., Sharma L., Yildirim A.Ö., Cruz C.S.D., Hogaboam C.M. (2018). Innate Immunity of the Lung: From Basic Mechanisms to Translational Medicine. J. Innate Immun..

[108] Berenson C.S., Kruzel R.L., Eberhardt E., Sethi S. (2013). Phagocytic Dysfunction of Human Alveolar Macrophages and Severity of Chronic Obstructive Pulmonary Disease. J. Infect. Dis..

[109] Lévêque M., Le Trionnaire S., Del Porto P., Martin-Chouly C. (2017). The impact of impaired macrophage functions in cystic fibrosis disease progression. J. Cyst. Fibros..

[110] Freeman C.M., Curtis J.L. (2017). Lung dendritic cells: Shaping immune responses throughout chronic obstructive pulmonary disease progression. Am. J. Respir. Cell Mol. Biol..

[111] Ni L., Dong C. (2018). Roles of Myeloid and Lymphoid Cells in the Pathogenesis of Chronic Obstructive Pulmonary Disease. Front. Immunol..

[112] David B., Bafadhel M., Koenderman L., De Soyza A. (2021). Eosinophilic inflammation in COPD: From an inflammatory marker to a treatable trait. Thorax.

[113] Borger J.G., Lau M., Hibbs M.L. (2019). The Influence of Innate Lymphoid Cells and Unconventional T Cells in Chronic Inflammatory Lung Disease. Front. Immunol..

[114] Vethanayagam R.R., Almyroudis N.G., Grimm M.J., Lewandowski D.C., Pham C.T.N., Blackwell T.S., Petraitiene R., Petraitis V., Walsh T.J., Urban C.F. (2011). Role of NADPH Oxidase versus Neutrophil Proteases in Antimicrobial Host Defense. PLoS ONE.

[115] Clancy D.M., Sullivan G.P., Moran H.B., Henry C.M., Reeves E.P., McElvaney N.G., Lavelle E.C., Martin S.J. (2018). Extracellular Neutrophil Proteases Are Efficient Regulators of IL-1, IL-33, and IL-36 Cytokine Activity but Poor Effectors of Microbial Killing. Cell Rep..

[116] Stapels D.A., Geisbrecht B.V., Rooijakkers S.H. (2015). Neutrophil serine proteases in antibacterial defense. Curr. Opin. Microbiol..

[117] Standish A.J., Weiser J.N. (2009). Human Neutrophils Kill Streptococcus pneumoniae via Serine Proteases. J. Immunol..

[118] Belaaouaj A., McCarthy R.T., Baumann M.L., Gao Z., Ley T.J., Abraham S.N., Shapiro S.D. (1998). Mice lacking neutrophil elastase reveal impaired host defense against gram negative bacterial sepsis. Nat. Med..

[119] Hirche T.O., Benabid R., Deslee G., Gangloff S., Achilefu S., Guenounou M., Lebargy F., Hancock R.E., Belaaouaj A. (2008). Neutrophil Elastase Mediates Innate Host Protection against Pseudomonas aeruginosa. J. Immunol..

[120] Müller S., Faulhaber A., Sieber C., Pfeifer D., Hochberg T., Gansz M., Deshmukh S.D., Dauth S., Brix K., Saftig P. (2013). The endolysosomal cysteine cathepsins L and K are involved in macrophage-mediated clearance of Staphylococcus aureus and the concomitant cytokine induction. FASEB J..

[121] Szulc-Dąbrowska L., Bossowska-Nowicka M., Struzik J., Toka F.N. (2020). Cathepsins in Bacteria-Macrophage Interaction: Defenders or Victims of Circumstance?. Front. Cell. Infect. Microbiol..

[122] Ewald S.E., Engel A., Lee J., Wang M., Bogyo M., Barton G.M. (2011). Nucleic acid recognition by Toll-like receptors is coupled to stepwise processing by cathepsins and asparagine endopeptidase. J. Exp. Med..

[123] Tsukuba T., Yamamoto S., Yanagawa M., Okamoto K., Okamoto Y., Nakayama K.I., Kadowaki T., Yamamoto K. (2006). Cathepsin E-deficient mice show in-creased susceptibility to bacterial infection associated with the decreased expression of multiple cell surface Toll-like receptors. J. Biochem..

[124] Hsieh C.-S., DeRoos P., Honey K., Beers C., Rudensky A.Y. (2002). A Role for Cathepsin L and Cathepsin S in Peptide Generation for MHC Class II Presentation. J. Immunol..

[125] Padrines M., Wolf M., Walz A., Baggiolini M. (1994). Interleukin-8 processing by neutrophil elastase, cathepsin G and proteinase-3. FEBS Lett..

[126] Nufer O., Corbett M., Walz A. (1999). Amino-Terminal Processing of Chemokine ENA-78 Regulates Biological Activity†. Biochemistry.

[127] Richter R., Bistrian R., Escher S., Forssmann W.-G., Vakili J., Henschler R., Spodsberg N., Frimpong-Boateng A., Forssmann U. (2005). Quantum Proteolytic Activation of Chemokine CCL15 by Neutrophil Granulocytes Modulates Mononuclear Cell Adhesiveness. J. Immunol..

[128] Wittamer V., Bondue B., Guillabert A., Vassart G., Parmentier M., Communi D. (2005). Neutrophil-Mediated Maturation of Chemerin: A Link between Innate and Adaptive Immunity. J. Immunol..

[129] Schonbeck U., Mach F., Libby P. (1998). Generation of biologically active IL-1 beta by matrix metalloproteinases: A novel caspa-se-1-independent pathway of IL-1 beta processing. J. Immunol..

[130] Lambrecht B.N., Vanderkerken M., Hammad H. (2018). The emerging role of ADAM metalloproteinases in immunity. Nat. Rev. Immunol..

[131] Gaggar A., Weathington N. (2016). Bioactive extracellular matrix fragments in lung health and disease. J. Clin. Investig..

[132] Turnbull A.R., Pyle C.J., Patel D.F., Jackson P.L., Hilliard T.N., Regamey N., Tan H.-L., Brown S., Thursfield R., Short C. (2019). Abnormal pro-gly-pro pathway and airway neu-trophilia in pediatric cystic fibrosis. J. Cyst. Fibros..

[133] Bonnans C., Chou J., Werb Z. (2014). Remodelling the extracellular matrix in development and disease. Nat. Rev. Mol. Cell Biol..

[134] Karakioulaki M., Papakonstantinou E., Stolz D. (2020). Extracellular matrix remodelling in COPD. Eur. Respir. Rev..

[135] Taggart C.C., Greene C.M., Smith S.G., Levine R.L., McCray P.B., O’Neill S., McElvaney N. (2003). Inactivation of human beta-defensins 2 and 3 by elastolytic cathepsins. J. Immunol..

[136] Andrault P.-M., Samsonov S.A., Weber G., Coquet L., Nazmi K., Bolscher J.G.M., Lalmanach A.-C., Jouenne T., Brömme D., Pisabarro M.T. (2015). Antimicrobial Peptide LL-37 Is Both a Substrate of Cathepsins S and K and a Selective Inhibitor of Cathepsin L. Biochemistry.

[137] Fischer B.M., Domowicz D.A.L., Zheng S., Carter J.L., McElvaney N.G., Taggart C., Lehmann J.R., Voynow J.A., Ghio A.J. (2009). Neutrophil elastase increases airway epi-thelial nonheme iron levels. Clin. Transl. Sci..

[138] Rubio F., Cooley J., Accurso F.J., Remold-O’Donnell E. (2004). Linkage of neutrophil serine proteases and decreased surfactant protein-A (SP-A) levels in inflammatory lung disease. Thorax.

[139] Cooley J., McDonald B., Accurso F.J., Crouch E.C., Remold-O’Donnell E. (2008). Patterns of neutrophil serine protease-dependent cleavage of surfactant protein D in inflammatory lung disease. J. Leukoc. Biol..

[140] Bratcher P.E., Weathington N.M., Nick H.J., Jackson P.L., Snelgrove R.J., Gaggar A. (2012). MMP-9 Cleaves SP-D and Abrogates Its Innate Immune Functions In Vitro. PLoS ONE.

[141] Han S., Mallampalli R.K. (2015). The Role of Surfactant in Lung Disease and Host Defense against Pulmonary Infections. Ann. Am. Thorac. Soc..

[142] Linden D., Guo-Parke H., Coyle P.V., Fairley D., McAuley D.F., Taggart C.C., Kidney J. (2019). Respiratory viral infection: A potential “missing link” in the pathogenesis of COPD. Eur. Respir. Rev..

[143] Böttcher-Friebertshäuser E., Freuer C., Sielaff F., Schmidt S., Eickmann M., Uhlendorff J., Steinmetzer T., Klenk H., Garten W. (2010). Cleavage of Influenza Virus Hemagglutinin by Airway Proteases TMPRSS2 and HAT Differs in Subcellular Localization and Susceptibility to Protease In-hibitors. J. Virol..

[144] Seth S., Batra J., Srinivasan S. (2020). COVID-19: Targeting Proteases in Viral Invasion and Host Immune Response. Front. Mol. Biosci..

[145] Simpson A., Maxwell A., Govan J., Haslett C., Sallenave J.-M. (1999). Elafin (elastase-specific inhibitor) has anti-microbial activity against Gram-positive and Gram-negative respiratory pathogens. FEBS Lett..

[146] Sallenave J.-M. (2010). Secretory Leukocyte Protease Inhibitor and Elafin/Trappin-2. Am. J. Respir. Cell Mol. Biol..

[147] Ries C. (2014). Cytokine functions of TIMP-1. Cell. Mol. Life Sci..

[148] Holloway A.J., Yu J., Arulanandam B.P., Hoskinson S.M., Eaves-Pyles T. (2017). Cystatins 9 and C as a Novel Immunotherapy Treatment That Protects against Multidrug-Resistant New Delhi Metallo-Beta-Lactamase-1-ProducingKlebsiella pneumoniae. Antimicrob. Agents Chemother..

[149] Guyot N., Butler M.W., McNally P., Weldon S., Greene C.M., Levine R.L., O’Neill S.J., Taggart C.C., McElvaney N.G. (2008). Elafin, an Elastase-specific Inhibitor, Is Cleaved by Its Cognate Enzyme Neutrophil Elastase in Sputum from Individuals with Cystic Fibrosis. J. Biol. Chem..

[150] Weldon S., McNally P., McElvaney N.G., Elborn J.S., McAuley D.F., Wartelle J., Belaaouaj A., Levine R.L., Taggart C.C. (2009). Decreased Levels of Secretory Leucoprotease Inhibitor in thePseudomonas-Infected Cystic Fibrosis Lung Are Due to Neutrophil Elastase Degradation. J. Immunol..

[151] Kerrin A., Weldon S., Chung A.H.-K., Craig T., Simpson A.J., O’Kane C.M., McAuley D.F., Taggart C.C. (2013). Proteolytic cleavage of elafin by 20S proteasome may contribute to inflammation in acute lung injury. Thorax.

[152] Taggart C.C., Lowe G.J., Greene C.M., Mulgrew A.T., O’Neill S.J., Levine R.L., McElvaney N.G. (2001). Cathepsin B, L, and S Cleave and Inactivate Secretory Leucoprotease Inhibitor. J. Biol. Chem..

[153] Johnson D.A., Barrettli A.J., Masonli R.W. (1986). Cathepsin L Inactivates Alpha 1-Proteinase Inhibitor by Cleavage in the Reactive Site Region. J. Biol. Chem..

[154] Liu Z., Zhou X., Shapiro S.D., Shipley J., Twining S.S., Diaz L.A., Senior R.M., Werb Z. (2000). The Serpin α1-Proteinase Inhibitor Is a Critical Substrate for Gelatinase B/MMP-9 In Vivo. Cell.

[155] Jackson P.L., Xu X., Wilson L., Weathington N.M., Clancy J.P., Blalock J.E., Gaggar A. (2010). Human Neutrophil Elastase-Mediated Cleavage Sites of MMP-9 and TIMP-1: Implications to Cystic Fibrosis Proteolytic Dysfunction. Mol. Med..

[156] Buttle D.J., Abrahamson M., Burnett D., Mort J.S., Barrett A.J., Dando P.M., Hill S.L. (1991). Human sputum cathepsin B degrades proteoglycan, is inhibited by α2-macroglobulin and is modulated by neutrophil elastase cleavage of cathepsin B precursor and cystatin C. Biochem. J..

[157] Dittrich A.S., Kühbandner I., Gehrig S., Rickert-Zacharias V., Twigg M., Wege S., Taggart C.C., Herth F., Schultz C., Mall M.A. (2018). Elastase activity on sputum neutrophils correlates with severity of lung disease in cystic fibrosis. Eur. Respir. J..

[158] Guerra M., Frey D., Hagner M., Dittrich S., Paulsen M., Mall M.A., Schultz C. (2019). Cathepsin G Activity as a New Marker for Detecting Airway Inflammation by Microscopy and Flow Cytometry. ACS Central Sci..

[159] Genschmer K.R., Russell D.W., Lal C., Szul T., Bratcher P.E., Noerager B.D., Roda M.A., Xu X., Rezonzew G., Viera L. (2019). Activated PMN Exosomes: Pathogenic Entities Causing Matrix Destruction and Disease in the Lung. Cell.

[160] Hu H.-Y., Gehrig S., Reither G., Subramanian D., Mall M.A., Plettenburg O., Schultz C. (2014). FRET-based and other fluorescent proteinase probes. Biotechnol. J..

[161] Hagner M., Frey D.L., Guerra M., Dittrich A.S., Halls V.S., Wege S., Herth F.J., Schultz C., Mall M.A. (2020). New method for rapid and dynamic quantification of elastase activity on sputum neutrophils from patients with cystic fibrosis using flow cytometry. Eur. Respir. J..

[162] Hamon Y., Legowska M., Hervé V., Dallet-Choisy S., Marchand-Adam S., Vanderlynden L., Demonte M., Williams R., Scott C.J., Si-Tahar M. (2016). Neutrophilic Cathepsin C Is Maturated by a Multistep Proteolytic Process and Secreted by Activated Cells during Inflammatory Lung Diseases. J. Biol. Chem..

[163] Méthot N., Rubin J., Guay D., Beaulieu C., Ethier D., Reddy T.J., Riendeau D., Percival M.D. (2007). Inhibition of the Activation of Multiple Serine Proteases with a Cathepsin C Inhibitor Requires Sustained Exposure to Prevent Pro-enzyme Processing. J. Biol. Chem..

[164] Garratt L.W., Sutanto E.N., Ling K.-M., Looi K., Iosifidis T., Martinovich K.M., Shaw N.C., Kicic-Starcevich E., Knight D.A., Ranganathan S. (2015). Matrix metalloproteinase activation by free neutrophil elastase contributes to bronchiectasis progression in early cystic fibrosis. Eur. Respir. J..

[165] Heuberger D.M., Schuepbach R.A. (2019). Protease-activated receptors (PARs): Mechanisms of action and potential therapeutic mod-ulators in PAR-driven inflammatory diseases. Thromb. J..

[166] Mercer P.F., Williams A.E., Scotton C.J., José R.J., Sulikowski M., Moffatt J.D., Murray L.A., Chambers R.C. (2013). Proteinase-Activated Receptor-1, CCL2 and CCL7 Regulate Acute Neutrophilic Lung Inflammation. Am. J. Respir. Cell Mol. Biol..

[167] José R.J., Williams A.E., Mercer P.F., Sulikowski M.G., Brown J.S., Chambers R.C. (2015). Regulation of Neutrophilic Inflammation by Pro-teinase-Activated Receptor 1 during Bacterial Pulmonary Infection. J. Immunol..

[168] Lin C., von der Thüsen J., Daalhuisen J., ten Brink M., Crestani B., van der Poll T., Borensztajn K., Spek C.A. (2015). Pharmacological Targeting of Prote-ase-Activated Receptor 2 Affords Protection from Bleomycin-Induced Pulmonary Fibrosis. Mol. Med..

[169] Asokananthan N., Graham P.T., Fink J., Knight D.A., Bakker A.J., McWilliam A.S., Thompson P.J., Stewart G.A. (2002). Activation of Protease-Activated Receptor (PAR)-1, PAR-2, and PAR-4 Stimulates IL-6, IL-8, and Prostaglandin E2Release from Human Respiratory Epithelial Cells. J. Immunol..

[170] Chambers R.C., Scotton C.J. (2012). Coagulation Cascade Proteinases in Lung Injury and Fibrosis. Proc. Am. Thorac. Soc..

[171] Small D.M., Brown R.R., Doherty D.F., Abladey A., Zhou-Suckow Z., Delaney R.J., Kerrigan L., Dougan C.M., Borensztajn K.S., Holsinger L. (2019). Targeting of cathepsin S reduces cystic fibrosis-like lung disease. Eur. Respir. J..

[172] Hou H.-H., Wang H.-C., Cheng S.-L., Chen Y.-F., Lu K.-Z., Yu C.-J. (2018). MMP-12 activates protease-activated receptor-1, upregulates placenta growth factor, and leads to pulmonary emphysema. Am. J. Physiol. Cell. Mol. Physiol..

[173] Suzuki T., Yamashita C., Zemans R.L., Briones N., Van Linden A., Downey G.P. (2009). Leukocyte elastase induces lung epithelial apoptosis via a PAR-1-, NF-kappaB-, and p53-dependent pathway. Am. J. Respir. Cell Mol. Biol..

[174] Kuliopulos A., Covic L., Seeley S.K., Sheridan P.J., Helin J., Costello C.E. (1999). Plasmin Desensitization of the PAR1 Thrombin Receptor: Kinetics, Sites of Truncation, and Implications for Thrombolytic Therapy†. Biochemistry.

[175] Wittekindt O.H. (2017). Tight junctions in pulmonary epithelia during lung inflammation. Pflügers Archiv-Eur. J. Physiol..

[176] Huguenin M., Müller E.J., Trachsel-Rösmann S., Oneda B., Ambort D., Sterchi E.E., Lottaz D. (2008). The Metalloprotease Meprinβ Processes E-Cadherin and Weakens Intercellular Adhesion. PLoS ONE.

[177] Bao J., Yura R.E., Matters G.L., Bradley S.G., Shi P., Tian F., Bond J.S. (2013). Meprin A impairs epithelial barrier function, enhances monocyte migration, and cleaves the tight junction protein occludin. Am. J. Physiol. Physiol..

[178] Brune K., Frank J.A., Schwingshackl A., Finigan J.H., Sidhaye V.K. (2015). Pulmonary epithelial barrier function: Some new players and mechanisms. Am. J. Physiol. Cell. Mol. Physiol..

[179] Churg A., Wang R., Wang X., Onnervik P.-O., Thim K., Wright J.L. (2007). Effect of an MMP-9/MMP-12 inhibitor on smoke-induced emphysema and airway remodelling in guinea pigs. Thorax.

[180] Janelle M.F., Doucet A., Bouchard D., Bourbonnais Y., Tremblay G.M. (2006). Increased local levels of granulocyte colony-stimulating factor are associated with the beneficial effect of pre-elafin (SKALP/trappin-2/WAP3) in experimental emphysema. Biol. Chem..

[181] Gehrig S., Duerr J., Weitnauer M., Wagner C.J., Graeber S.Y., Schatterny J., Hirtz S., Belaaouaj A., Dalpke A.H., Schultz C. (2014). Lack of Neutrophil Elastase Reduces Inflammation, Mucus Hypersecretion, and Emphysema, but Not Mucus Obstruction, in Mice with Cystic Fibrosis–like Lung Disease. Am. J. Respir. Crit. Care Med..

[182] Dirksen A., Piitulainen E., Parr D.G., Deng C., Wencker M., Shaker S.B., Stockleye R.A. (2009). Exploring the role of CT densitometry: A randomised study of augmentation therapy in α1-antitrypsin deficiency. Eur. Respir. J..

[183] Chapman K.R., Burdon J.G.W., Piitulainen E., Sandhaus R.A., Seersholm N., Stocks J.M., Stoel B.C., Huang L., Yao Z., Edelman J.M. (2015). Intravenous augmentation treatment and lung density in severe α1 antitrypsin deficiency (RAPID): A randomised, double-blind, placebo-controlled trial. Lancet.

[184] McElvaney N.G., Burdon J., Holmes M., Glanville A., Wark P.A.B., Thompson P.J., Hernandez P., Chlumsky J., Teschler H., Ficker J.H. (2017). Long-term efficacy and safety of α1 proteinase inhibitor treatment for emphysema caused by severe α1 antitrypsin deficiency: An open-label extension trial (RAP-ID-OLE). Lancet Respir. Med..

[185] Owen C.A., Campbell M.A., Sannes P.L., Boukedes S.S., Campbell E.J. (1995). Cell surface-bound elastase and cathepsin G on human neutrophils: A novel, non-oxidative mechanism by which neutrophils focus and preserve catalytic activity of serine proteinases. J. Cell Biol..

[186] Owen C.A., Hu Z., Barrick B., Shapiro S.D. (2003). Inducible expression of tissue inhibitor of metalloproteinases-resistant matrix metal-loproteinase-9 on the cell surface of neutrophils. Am. J. Respir. Cell Mol. Biol..

[187] Allan E.R.O., Yates R.M. (2015). Redundancy between Cysteine Cathepsins in Murine Experimental Autoimmune Encephalomyelitis. PLoS ONE.

[188] Vandenbroucke R.E., Libert C. (2014). Is there new hope for therapeutic matrix metalloproteinase inhibition?. Nat. Rev. Drug Discov..

[189] Griese M., Latzin P., Kappler M., Weckerle K., Heinzimaier T., Bernhardt T., Hartl D. (2007). α1-Antitrypsin inhalation reduces airway inflammation in cystic fibrosis patients. Eur. Respir. J..

[190] Phase II Study of the Safety and Efficacy of Inhaled Alpha-1 Antitrypsin (AAT) in Cystic Fibrosis Patients-Full Text View-ClinicalTrials.gov. https://www.clinicaltrials.gov/ct2/show/NCT00499837.

[191] Inhaled A1AT in Adult Stable Bronchiectasis-Full Text View-ClinicalTrials.gov. https://clinicaltrials.gov/ct2/show/NCT03383939.

[192] Campos M.A., Geraghty P., Holt G., Mendes E., Newby P.R., Ma S., Luna-Diaz L.V., Turino G.M., Stockley R.A. (2019). The Biological Effects of Double-Dose Alpha-1 Antitrypsin Augmentation Therapy. A Pilot Clinical Trial. Am. J. Respir. Crit. Care Med..

[193] Phase II, Safety and Efficacy Study of Kamada-alpha-1-antitrypsin (AAT) for Inhalation-Study Results-ClinicalTrials.gov. https://clinicaltrials.gov/ct2/show/NCT02001688.

[194] Stage 1 Study of ARALAST NP and GLASSIA in A1PI Deficiency-Study Results-ClinicalTrials.gov. https://clinicaltrials.gov/ct2/show/NCT02722304.

[195] Elborn J.S., Perrett J., Forsman-Semb K., Marks-Konczalik J., Gunawardena K., Entwistle N. (2012). Efficacy, safety and effect on biomarkers of AZD9668 in cystic fibrosis. Eur. Respir. J..

[196] Kuna P., Jenkins M., O’Brien C.D., Fahy W.A. (2012). AZD9668, a neutrophil elastase inhibitor, plus ongoing budesonide/ formoterol in patients with COPD. Respir. Med..

[197] Vogelmeier C., Aquino T.O., O’Brien C.D., Perrett J., Gunawardena K.A. (2012). A randomised, placebo-controlled, dose-finding study of AZD9668, an oral inhibitor of neutrophil elastase, in patients with chronic obstructive pulmonary disease treated with tiotropium. COPD J. Chronic Obstr. Pulm. Dis..

[198] Stockley R., De Soyza A., Gunawardena K., Perrett J., Forsman-Semb K., Entwistle N., Snell N. (2013). Phase II study of a neutrophil elastase inhibitor (AZD9668) in patients with bronchiectasis. Respir. Med..

[199] A Study to Assess Safety, Tolerability and Pharmacokinetics of of AZD6553 in Healthy Volunteers and Patients with Chronic Obstructive Pulmonary Disease (COPD)-Full Text View-ClinicalTrials.gov. https://clinicaltrials.gov/ct2/show/NCT01068184.

[200] A 12-Week Study Treating Participants Who Have Alpha1-Antitrypsin-Related COPD with Alvelestat (MPH966) or Placebo. Full Text View-ClinicalTrials.gov. https://www.clinicaltrials.gov/ct2/show/NCT03636347.

[201] Barth P., Bruijnzeel P., Wach A., Kessler O.S., Hooftman L., Zimmermann J., Naue N., Huber B., Heimbeck I., Kappeler D. (2020). Single dose escalation studies with inhaled POL6014, a potent novel selective reversible inhibitor of human neutrophil elastase, in healthy volunteers and subjects with cystic fibrosis. J. Cyst. Fibros..

[202] Watz H., Pedersen F., Kirsten A., Nagelschmitz J., Bandel T.-J., Schwers S., Rabe K. (2016). Safety and tolerability of the NE inhibitor BAY 85-8501 in patients with non-CF bronchiectasis. Eur. Respir. J..

[203] Clinical Study to Investigate Safety, Tolerability, Pharmacokinetics and Pharmacodynamics of POL6014 in Patients with CF Full Text View-ClinicalTrials.gov. https://clinicaltrials.gov/ct2/show/NCT03748199.

[204] A Study to Investigate Safety, Tolerability and Pharmacokinetics of Single and Repeat Doses of CHF6333 in Healthy Subjects-Full Text View-ClinicalTrials.gov. https://clinicaltrials.gov/ct2/show/NCT03056326.

[205] A Study in Patients with Non-cystic Fibrosis Bronchiectasis to Test How Well Different Doses of BI 1323495 Are Tolerated and How BI 1323495 Affects Biomarkers of Inflammation-Full Text View-ClinicalTrials.gov. https://clinicaltrials.gov/ct2/show/NCT04656275.

[206] Watz H., Nagelschmitz J., Kirsten A., Pedersen F., van der Mey D., Schwers S., Bandel T.-J., Rabe K.F. (2019). Safety and efficacy of the human neutrophil elastase inhibitor BAY 85-8501 for the treatment of non-cystic fibrosis bronchiectasis: A randomized controlled trial. Pulm. Pharmacol. Ther..

[207] Dahl R., Titlestad I., Lindqvist A., Wielders P., Wray H., Wang M., Samuelsson V., Mo J., Holt A. (2012). Effects of an oral MMP-9 and -12 inhibitor, AZD1236, on biomarkers in moderate/severe COPD: A randomised controlled trial. Pulm. Pharmacol. Ther..

[208] Chalmers J.D., Haworth C.S., Metersky M.L., Loebinger M.R., Blasi F., Sibila O., O’Donnell A.E., Sullivan E.J., Mange K.C., Fernandez C. (2020). Phase 2 Trial of the DPP-1 Inhibitor Brensocatib in Bronchiectasis. N. Engl. J. Med..

[209] Geraghty P., Rogan M.P., Greene C.M., Boxio R.M.M., Poiriert T., O’Mahony M., Belaaouaj A., O’Neill S.J., Taggart C.C., McElvaney N.G. (2007). Neutrophil elastase up-regulates cathepsin B and matrix metalloprotease-2 expression. J. Immunol..

[210] Geraghty P., Rogan M.P., Greene C.M., Brantly M.L., O’Neill S.J., Taggart C.C., McElvaney N.G. (2008). Alpha-1-antitrypsin aerosolised augmentation abrogates neutrophil elastase-induced expression of cathepsin B and matrix metalloprotease 2 in vivo and in vitro. Thorax.

[211] O’Donnell A.E., Barker A.F., Ilowite J.S., Fick R.B. (1998). Treatment of Idiopathic Bronchiectasis with Aerosolized Recombinant Human DNase I. Chest.

[212] Fuchs H.J., Borowitz D.S., Christiansen D.H., Morris E.M., Nash M.L., Ramsey B.W., Rosenstein B.J., Smith A.L., Wohl M.E. (1994). Effect of Aerosolized Recombinant Human DNase on Exacerbations of Respiratory Symptoms and on Pulmonary Function in Patients with Cystic Fibrosis. N. Engl. J. Med..

[213] Decramer M., Rutten-Van Mölken M., Dekhuijzen P.N.R., Troosters T., Van Herwaarden C., Pellegrino R., Van Schayk C.P., Olivieri D., Del Donno M., De Backer W. (2005). Effects of N-acetylcysteine on outcomes in chronic obstructive pulmonary disease (Bronchitis Randomized on NAC Cost-Utility Study, BRONCUS): A randomised placebo-controlled trial. Lancet.

[214] Dal Negro R.W., Wedzicha J.A., Iversen M., Fontana G., Page C., Cicero A.F., Pozzi E., Calverley P.M.A. (2017). Effect of erdosteine on the rate and duration of COPD exacerbations: The RESTORE study. Eur. Respir. J..

[215] Aaron S.D. (2017). Mucolytics for COPD: Negotiating a slippery slope towards proof of efficacy. Eur. Respir. J..

[216] Leal J., Smyth H.D., Ghosh D. (2017). Physicochemical properties of mucus and their impact on transmucosal drug delivery. Int. J. Pharm..

